# Investigation of erythema migrans patients identifies *Borrelia* species and *Neoehrlichia mikurensis* with implications for clinical assessment

**DOI:** 10.1038/s41598-025-07291-0

**Published:** 2025-06-25

**Authors:** Samuel Cronhjort, Peter Wilhelmsson, Ivar Tjernberg, Mattias Waldeck, Anna J. Henningsson, Chris Anderson, Pia Forsberg, Per-Eric Lindgren

**Affiliations:** 1 Division of Inflammation and Infection, Department of Biomedical and Clinical Sciences, Linköping University, Linköping, Sweden; 2 Department of Clinical Chemistry and Transfusion Medicine, Region Kalmar County, Kalmar, Sweden; 3 Regional Office of Communicable Disease Control and Prevention, Malmö, Region Skåne Sweden; 4 Division of Clinical Microbiology, Department of Laboratory Medicine, Region Jönköping County, Jönköping, Sweden; 5 Division of Cell and Neurobiology, Department of Biomedical and Clinical Sciences, Linköping University, Linköping, Sweden

**Keywords:** Infectious-disease diagnostics, Pathogens

## Abstract

Emerging tick-borne infections pose public health challenges and may complicate treatment decisions. The EMBio study, a multicenter observational study, aims to describe erythema migrans (EM), an early localized manifestation of *Borrelia burgdorferi sensu lato* (s.l.) infection, and investigate the occurrence of tick-borne co-infections among patients presenting with this skin lesion. Additionally, the study seeks to determine relations between EM morphology, other clinical manifestations, specific pathogens, and disease prognosis. Clinical characteristics, skin biopsies, and blood samples were analyzed from 26 patients to assess co-infections, quantity, *Borrelia* species, and spirochete load. *Borrelia* DNA was detected in 88% of EM skin lesions, with *Borrelia afzelii* as the predominant species. Two cases of co-infections were identified, one involving two *Borrelia* species and one involving *Borrelia afzelii and* the intracellular bacterium *Neoehrlichia mikurensis*. Notably, homogeneous EM lesions harbored significantly higher spirochete quantities in the central zone compared to annular lesions, suggesting that lesion morphology reflects local bacterial density. This supports the value of molecular diagnostics in detecting mixed infections and supports morphology-guided biopsy strategies in the clinical assessment of cutaneous infections. This study contributes to a better understanding of co-infection dynamics and may improve diagnostic accuracy and patient management in endemic settings.

**Supplementary Information:**

The online version contains supplementary material available at 10.1038/s41598-025-07291-0.

## Introduction

Tick-borne human pathogens are highly prevalent in ticks from Sweden^1^. Ticks can carry multiple pathogens, which may be simultaneously transmitted to humans through tick bites. The EMBio study, a multicenter observational study, aims to describe erythema migrans (EM), an early localized manifestation of *Borrelia burgdorferi* sensu lato (s.l.) infection, and investigate the occurrence of tick-borne co-infections among patients presenting with this skin lesion. Additionally, the study seeks to determine relations between EM morphology, other clinical manifestations, specific pathogens, and disease prognosis.

Two out of 26 EM patients had co-infections (8%, Table 1) detected by PCR. One patient had co-infection with *B. afzelii* and *B. garinii*, both detectable by PCR in the obtained skin biopsies. The patient had a homogeneous EM on one leg and reported fatigue and vertigo upon inclusion in the study. The co-infection was suspected due to multiple signals in the electropherogram and confirmed through RipSeq Mixed sequence analysis. Only *B. garinii* was detected in the central zone of the EM, whereas both *B. afzelii* and *B. garinii* were detected in the border zone. Another patient, who had an EM on the abdomen and tested positive for *B. afzelii* in the skin biopsy, was also positive for *Neoehrlichia mikurensis* in the blood at the time of study inclusion. This patient did not report any clinical symptoms except the EM. PCR positivity for *N. mikurensis* did not persist at the one-month follow-up.

Twenty-four patients completed the one-month follow-up. No patients reported fever. Other symptoms and characteristics are presented in Table 1. Eleven patients were aware of a tick bite preceding the appearance of EM, and eight of them estimated that they removed the tick within 24 h. The EM were mostly (14 of 26) located to the lower extremities. The EM were of mixed morphology, 11 were annular (with central clearing) and 11 homogeneous (without central clearing). Morphology could not be assessed in four patients due to missing photography or allergic reaction to the local anesthetic. Twelve patients reported that they had previously been diagnosed with LB, and one reported previous Human Granulocytic Anaplasmosis. Thirteen patients reported no previous tick-borne infection.

Twelve patients were seropositive for *B. burgdorferi* s. l. IgG (VlsE) antibodies at the time of inclusion in the study, and six patients were seropositive for IgM (VlsE, OspC) antibodies. Interestingly, only one patient seroconverted in IgM at the one-month follow-up, and only two patients seroconverted in IgG. *Borrelia* DNA was detected in skin biopsies taken from the EM in 23 out of 26 patients. *B. afzelii* was identified in fifteen patients, and *B. garinii* in two patients. *Borrelia* species could not be determined for the remaining seven patients with detected *Borrelia* DNA as nested PCR did not produce a product of expected length or readable sequence. The patients with the highest number of spirochetes were also IgG seropositive by inclusion in the study, confirming lack of protective effect from VlsE-specific IgG. This was further confirmed by correlation analyses, where levels of neither IgM nor IgG were negatively correlated to the number of spirochetes in the biopsies (IgM: r_s_ = 0.25, 95% CI -0.15 to 0.58; IgG: r_s_ = 0.023, 95% CI -0.37 to 0.41).

**Fig. 1:**
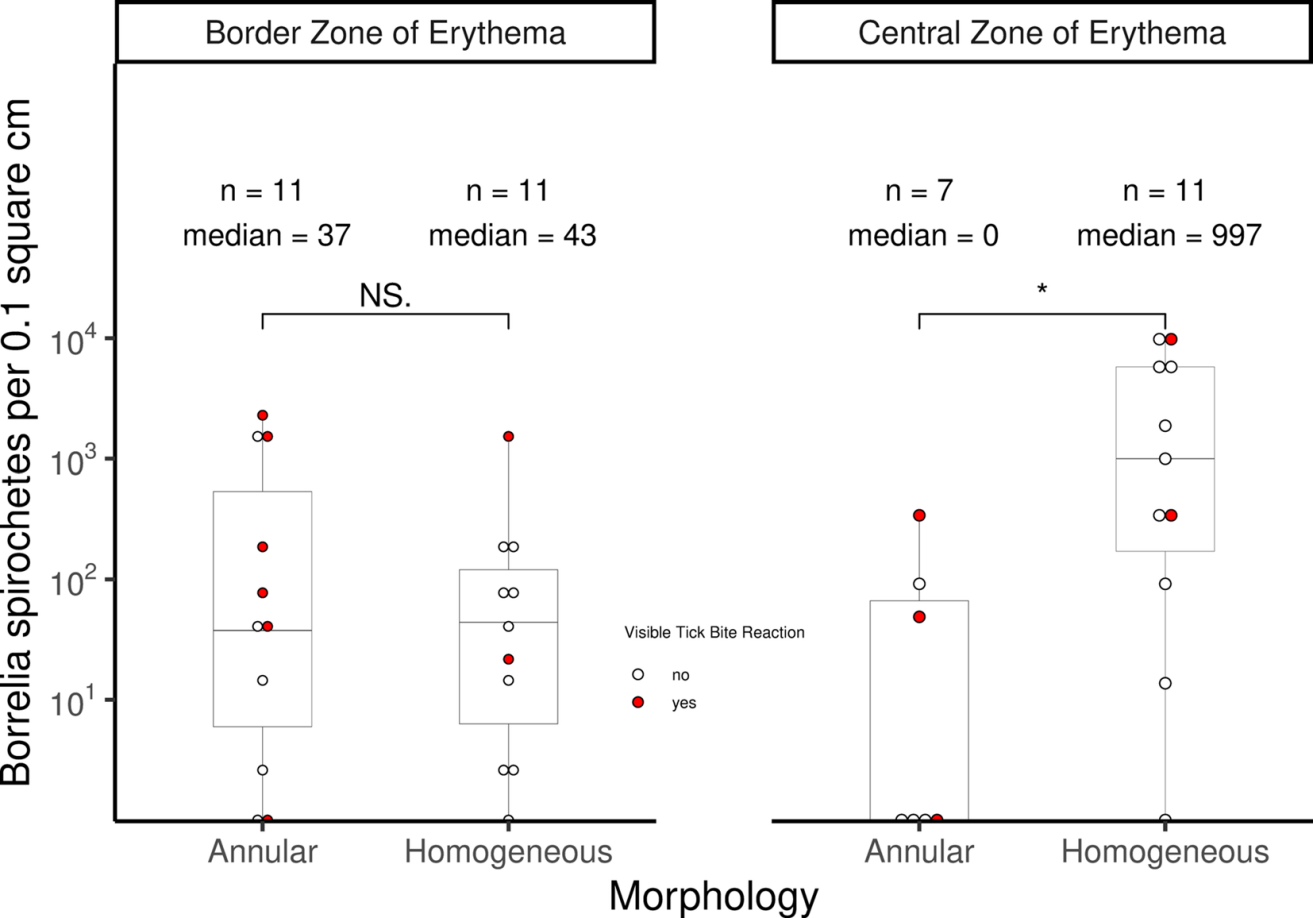
Boxplot of spirochete counts by biopsy location and morphology. Box limits are interquartile range, and center line is median. Whiskers represent 1.5x IQR. Homogeneous EM lesions contain a higher number of spirochetes in the central zone compared to annular EM lesions (Two-sided Wilcoxon rank sum test: *p* = 0.014, 95% CI 12.7 to 6134). Dots colorized red if there was a visible tick bite reaction within the EM.

Homogeneous EM had higher numbers of *Borrelia* spirochetes in the central zone compared to annular EM (Fig. 1, two-sided Wilcoxon rank sum test, *p* = 0.014, 95% CI 12.7 to 6134). This information may assist clinicians in determining the most appropriate biopsy location for detecting *Borrelia* DNA by PCR, particularly in cases of diagnostic uncertainty. We suggest sampling from the border zone of annular EM, and both central and border zones of homogeneous EM. Results from previous studies^2–4^in which biopsies were only from the border zone of EM, have concluded that annular EM, compared to homogeneous EM, have a higher burden of *Borrelia* spirochetes, but this could not be confirmed in the present study.

The correlation coefficient between diameter and patient’s delay was *r*_*s*_ 0.59 (*p* = 0.0058, 95% CI 0.17 to 0.83) (Fig. S1). The velocity of spirochetal migration in vivo is uncertain, yet crucial to understand concerning the course of the infection ranging from tick bite to disseminated infection. Expansion of EM was estimated by two different linear models based on time elapsed from discovery of the EM until inclusion in the study. The first linear regression model, excluding samples with negative *Borrelia* PCR, resulted in a co-efficient of 0.085, which suggests that EM expands by around 0.85 mm per day after discovery of the EM. The second model also excluded outliers with a longer patient’s delay than four weeks due to the risk of recall bias, this resulted in an estimate of around 3.8 mm migration velocity per day. Estimates from these clinical cases are uncertain due to unreliability of self-reported estimates and the low sample size, and therefore require further confirmation in larger studies. Previous in vitro work by Moriarty^5^ estimated that the rate of spirochetal extravasation is 3.4 μm/min, which can be extrapolated to 4.9 mm per 24 h. If we assume that spirochetal extravasation is relevant for transmission or movement in skin, this may be compared to our estimates. The rate of extravasation is higher than the estimates in this study, however estimates may vary due to differences between *Borrelia* species, as well as various in vivo factors and uncertainty due to our small sample size.

Our data (Fig. S2) does not show a clear correlation between patient sex and EM morphology. Unfortunately, we have too few confirmed cases of *B. garinii* infection to assess the impact of species. Previous studies in the Nordic countries^6,7^ on EM have suggested that women and *B. garinii* infections are more likely to be associated with homogeneous EM. Since there is a clear difference in number of spirochetes based on EM morphology and location of the biopsy within the EM – we hypothesize that the morphology is a consequence of the distribution of occurring number of spirochetes rather than the host’s sex.

In conclusion, EM following a tick bite in Sweden indicates an on-going *Borrelia* infection, which may involve multiple simultaneous species of *Borrelia*. There is also the possibility of infection by other tick-borne pathogens such as *N. mikurensis*. The significance of *N. mikurensis* and other tick-borne pathogens such as *Anaplasma phagocytophilum* or *Rickettsia helvetica* in EM patients remains uncertain, and their implications for treatment and clinical outcome require further study. In a clinical setting where confirmation of *Borrelia* diagnosis is necessary, our results indicate that a biopsy for analysis of *Borrelia* DNA should be taken from the border zone of annular EM and either the central area or both central and border zones of homogeneous EM to successfully detect *Borrelia* DNA. VlsE-specific immunoglobulins do not appear to reduce the number of spirochetes in the skin.

## Methods

### Study design

The diagnostic criterium for EM in Sweden is an expanding red or blue-red skin lesion (≥ 5 cm in diameter) with or without central clearing. The study design is summarized in Fig. S3. Adult patients capable of informed consent with clinical diagnosis of EM from participating outpatient clinics were considered eligible. Patients in whom a biopsy was contraindicated (such as known allergies to local anesthetics, or inappropriate localization of the EM for a biopsy) by the physician were excluded. All patients were treated according to standard care. Geographical bias was addressed through a multicenter design, and eligible patients were included after written informed consent at nine outpatient clinics (Fig. S4) in South-Eastern Sweden during 2018–2022. At the time of inclusion, physicians and patients completed questionnaires (S5) containing clinical data, self-reported symptoms and, if applicable, date and location of the tick bite. The EM was photographed. One to three biopsies were taken. Patients were included in the study even if they only consented to a single biopsy. The three punch biopsies were, in order of priority: Outer border of EM (4 mm), visually unaffected skin outside the EM (2 mm), and center of EM or visible tick-bite reaction (2 mm). Biopsies were immediately submerged in RNALater (Thermo-Fisher Scientific, USA), and sent by mail to the National Reference Laboratory (NRL) for *Borrelia* and other tick-borne bacteria (NRL) at the Department of Clinical Microbiology, County Hospital Ryhov, Jönköping, Sweden, and upon arrival frozen at − 20 °C. Blood and serum samples were also acquired from the patient, and frozen at − 70 °C. After one month, patients received another questionnaire by mail and complementary blood samples were acquired.

### Ethical statement

The study was approved by the Swedish Ethical Review Authority (Linköping: 2017/486 − 31, 2018/310 − 32) and conducted in accordance with the Declaration of Helsinki and local guidelines and regulations. All patients were included in the study after written informed consent. Human biological samples are stored at the Biobank facility at County Hospital Ryhov, Jönköping, Sweden according to the Swedish Biobank Act (2023:38).

### PCR detection methods

Detection of *Borrelia* spp., *Rickettsia* spp., *Babesia* spp., *Neoehrlichia mikurensis*,* Anaplasma phagocytophilum*, and relapsing fever *Borrelia miyamotoi* by real-time PCR was performed on biopsies and blood samples. Total nucleic acids were extracted using EZ1 RNA Tissue Mini Kit according to supplementary protocol “Isolation of total nucleic acids from animal and human tissues using the EZ1 RNA Tissue Mini Kit” (Qiagen, Kista, Sweden). Complementary DNA synthesis was performed using Illustra™ Ready-to-Go RT-PCR Beads kit (GE Healthcare, Chicago, USA) according to the manufacturer’s instructions. *Borrelia afzelii* was extracted from culture at a known concentration to enable real-time PCR-based quantification ex vivo. Number of spirochetes in biopsies were estimated from ct-values from the real-time PCR and normalized by area of the punch biopsy. Mixed electropherogram with suspected co-infection infection were analyzed using RipSeq Mixed web application (http://www.ripseq.com/) (Pathogenomix, Santa Cruz, California, USA). Probe-based assays utilized Maxima Probe qPCR Master Mix (Thermo-Fisher Scientific), and SYBR Green assays utilized Maxima SYBR Green Master Mix (Thermo-Fisher Scientific).

Detection of *Borrelia* spp. was analyzed by real-time PCR targeting a 116 bp amplicon of *16 S* rRNA, as previously described by Gyllemark et al.^8^. The primers used were: forward - GCT GAG TCA CGA AAG CGT AG, reverse - CAC TTA ACA CGT TAG CTT CGG TA, and the probe FAM-CGC TGT AAA CGA TGC ACA CTT GGT-MGB. The thermal cycling protocol consisted of an initial denaturation at 95° C for 5 min, followed by 50 cycles of 95° C for 10 s and 60° C for 60 s.

Genospecies identification in PCR-positive samples was carried out by nucleotide sequencing (Macrogen Inc., Amsterdam, the Netherlands) of amplicons generated by nested PCR. This assay targeted the intergenic spacer region (IGS) between *5 S* and *23 S* rRNA genes, following the method described by Wilhelmsson et al.^9^.

Samples that did not produce an amplicon of expected length or failed to yield a readable sequence from the IGS region were further analyzed by nested PCR of MLST housekeeping genes as previously described by Margos et al.^10^.

Samples in which the species could not be determined through sequencing of either IGS region or MLST genes were classified as *Borrelia* spp., without species-level identification.

Detection of *B. miyamotoi* was analyzed by a species-specific real-time PCR targeting a 156 bp amplicon of *flaB* gene, previously described by Hovius et al.^11^. The primers used were: forward - AGA AGG TGC TCA AGC AG, reverse - TCG ATC TTT GAA AGT GAC ATA T, and the probe FAM-AGC ACA GGA GGG AGT TCA AGC-BHQ1. The thermal cycling protocol consisted of an initial denaturation at 95° C for 10 min followed by 45 cycles of 95° C for 5 s and 60° C for 35 s.

Detection of *A. phagocytophilum* was analyzed by a species-specific real-time PCR targeting a 64 bp amplicon of *gltA* gene, as previously described by Henningsson et al.^12^. Primers used were: forward - TTT TGG GCG CTG AAT ACG AT, reverse - TCT CGA GGG AAT GAT CTA ATA ACG T, and the probe FAM-TGC CTG AAC AAG TTA TG-BHQ1. The thermal cycling protocol consisted of an initial denaturation at 95° C for 2 min, followed by 45 cycles of 95° C for 30 s and 60° C for 1 min.

Detection of *Rickettsia* spp. was analyzed by real-time PCR targeting a 75 bp amplicon of *gltA* gene, as previously described by Stenos et al.^13^. Primers used were: forward - TCG CAA ATG TTC ACG GTA CTT T, reverse - TCG TGC ATT TCT TTC CAT TGT G, and the probe FAM-TGC AAT AGC AAG AAC CGT AGG CTG GAT G-BHQ1. The thermal cycling protocol consisted of 3 min of 50° C, 5 min of 95° C, followed by 60 cycles of 95° C for 20 s and 60° C for 40 s.

Detection of *N. mikurensis* was analyzed by real-time SYBR green PCR targeting a 106 bp amplicon of *16 S* rRNA gene, as previously described by Labbé Sandelin et al.^14^. The primers used were: forward - GTA AAG GGC ATG TAG GCG GTT TAA, and reverse - TCC ACT ATC CTC TCT CGA TCT CTA GTT TAA. The thermal cycling protocol consisted of an initial denaturation at 95° C for 3 min, followed by 60 cycles of 95° C for 15 s, 60° C for 30 s, and 72° C for 30 s, followed by melting curve analysis.

Detection of *Babesia* spp. was analyzed by real-time SYBR green PCR targeting a 411 to 452 bp region of *18 S* rRNA gene, as previously described by Wilhelmsson et al.^15^. The primers used were: forward - GTC TTG TAA TTG GAA TGA TGG, and reverse - TAG TTT ATG GTT AGG ACT ACG. The thermal cycling protocol consisted of an initial denaturation at 94° C for 10 min, followed by 35 cycles of 94° C for 1 min, 55° C for 1 min, and 72° C for 2 min. The protocol concluded with a final extension at 72° C for 5 min, followed by melting curve analysis.

### Serological assays

Anti-*Borrelia* IgM was assessed with recombinant VlsE and OspC antigen (VirClia, Vircell, Granada, Spain), and positive results in IgM were confirmed by VlsE and OspC antigens immunoblot (Anti-*Borrelia* EUROLINE-RN-AT IgM, EUROIMMUN, Lübeck, Germany) from *B. afzelii*,* B. garinii* and *B. burgdorferi* sensu stricto (s.s.). Anti-*Borrelia* IgG was assessed with recombinant VlsE antigens (Liaison, DiaSorin, Saluggia, Italy). Serological analyses were performed at the NRL in Jönköping using assays that were part of the clinical routine and conducted according to manufacturer’s instructions.

**Table 1:**
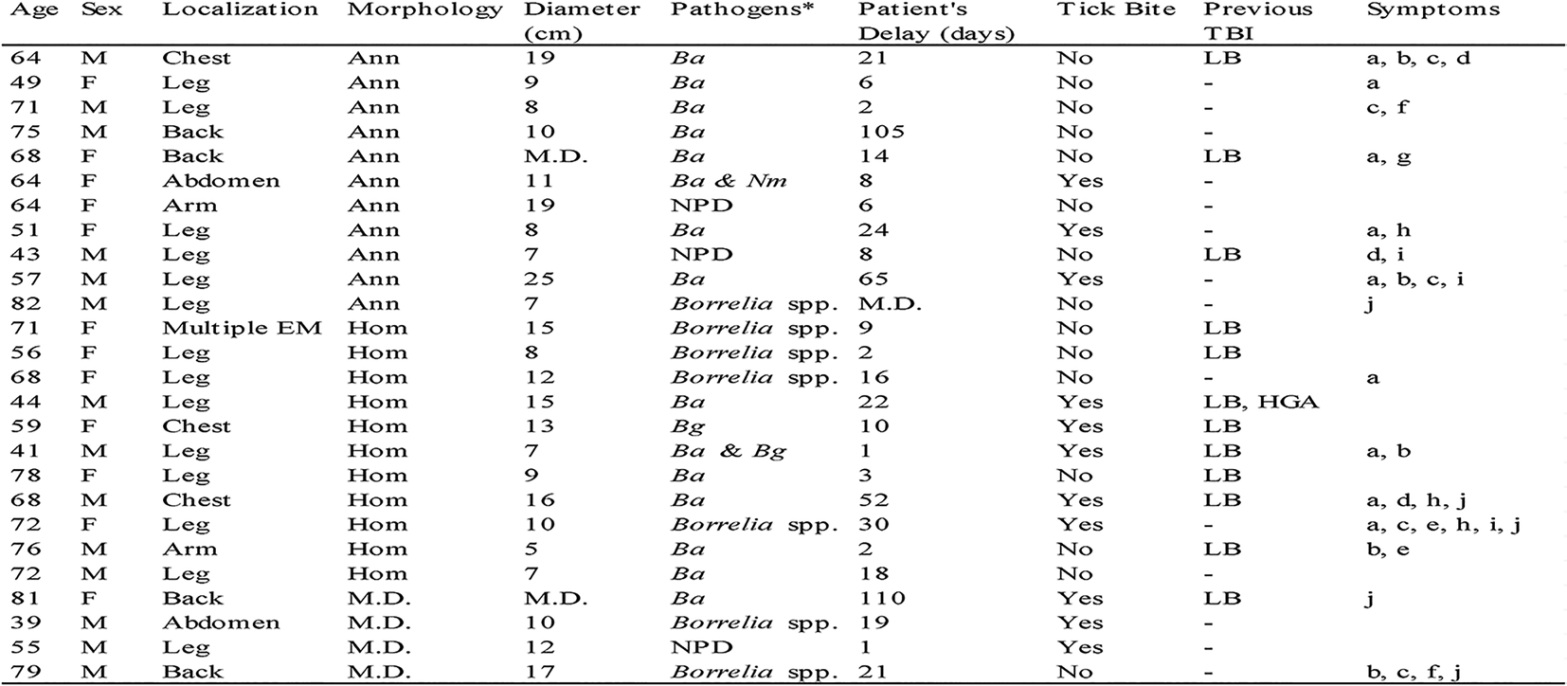
Patient characteristics. *EM* erythema migrans, *Ann* annular, *Hom* homogeneous, *M. D.* Missing data, *Ba Borrelia afzelii*, *Bg Borrelia garinii*, *Nm Neoehrlichia mikurensis*, *LB* Lyme borreliosis, *NPD* no pathogen detected, *HGA* human granulocytic anaplasmosis, *TBI* Tick-borne infection. Self-reported symptoms: (a) fatigue, (b) vertigo, (c) deficit attention, (d) paraesthesia, (e) neck stiffness, (f) radiating pains, (g) weight loss, (h) headache, (i) sono-photophobia, (j) myalgia. *Borrelia species were detected in skin biopsies, *N. mikurensis* was detected in blood. All other tested pathogens in skin and blood were considered negative by real-time PCR.

### Assessment of photographs and clinical data

Photographs were assessed by SC according to a predetermined protocol (S6) developed by dermatologist and Professor CA. Clinical data was extracted from questionnaires. Patient’s delay was defined as the time elapsed, in days, between when the patient discovered the rash, until inclusion in the study.

### Statistics

Data normality was estimated using the Shapiro-Wilk test. Correlations were estimated using Spearman rank correlation coefficient. Estimation of EM’s expansion was performed with two different linear regression models (Fig. S1), one excluding *Borrelia* PCR negative patients, and one also excluding study participants with extended patient’s delay (> 28 days). The two-sided Wilcoxon rank sum test was used to assess the difference between groups. Participants with missing data were excluded from analyses where applicable (Table 1). Statistical analyses were performed in the R statistical language^16^and plots were created using Tidyverse^17^. A p-value < 0.05 was considered significant.

## Electronic supplementary material

Below is the link to the electronic supplementary material.

Supplementary Material 1

## Data Availability

Data is available online by DOI 10.6084/m9.figshare.28614677.
