# Supplementary material for: Investigation of erythema migrans patients identifies Borrelia species and Neoehrlichia mikurensis with implications for clinical assessment
Source: Sci Rep. 2025 Jun 25;15:20293. doi: 10.1038/s41598-025-07291-0 (PMC12198406; doi:10.1038/s41598-025-07291-0)
Supplement: Supplementary file 1 — Supplementary Material 1 [file 41598_2025_7291_MOESM1_ESM.docx]

# Extended data


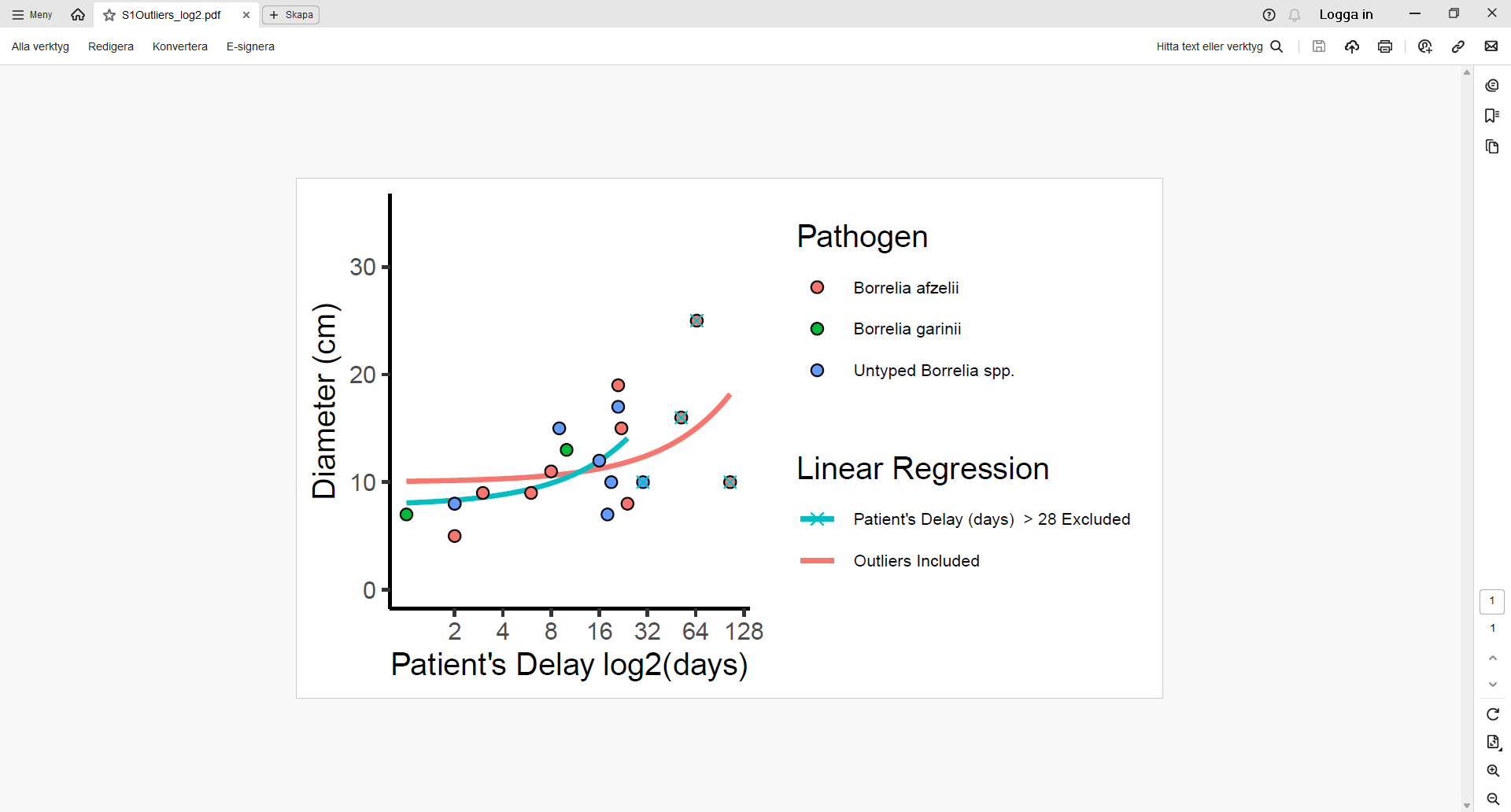


## Figure S1. Scatter Plot of Erythema Migrans Diameter vs. Patient’s delay, Color-Coded by Borrelia species (n=19) The scatter plot shows the relationship between the diameter of erythema migrans lesions, and the number of days elapsed since the patient discovered the rash, and inclusion in the study. The Spearman correlation coefficient is 0.59 (p = 0.0058, 95% CI 0.17 to 0.83). Linear regression models suggest that erythema migrans lesions expand by an average of 0.85 to 3.8 mm per day.

**
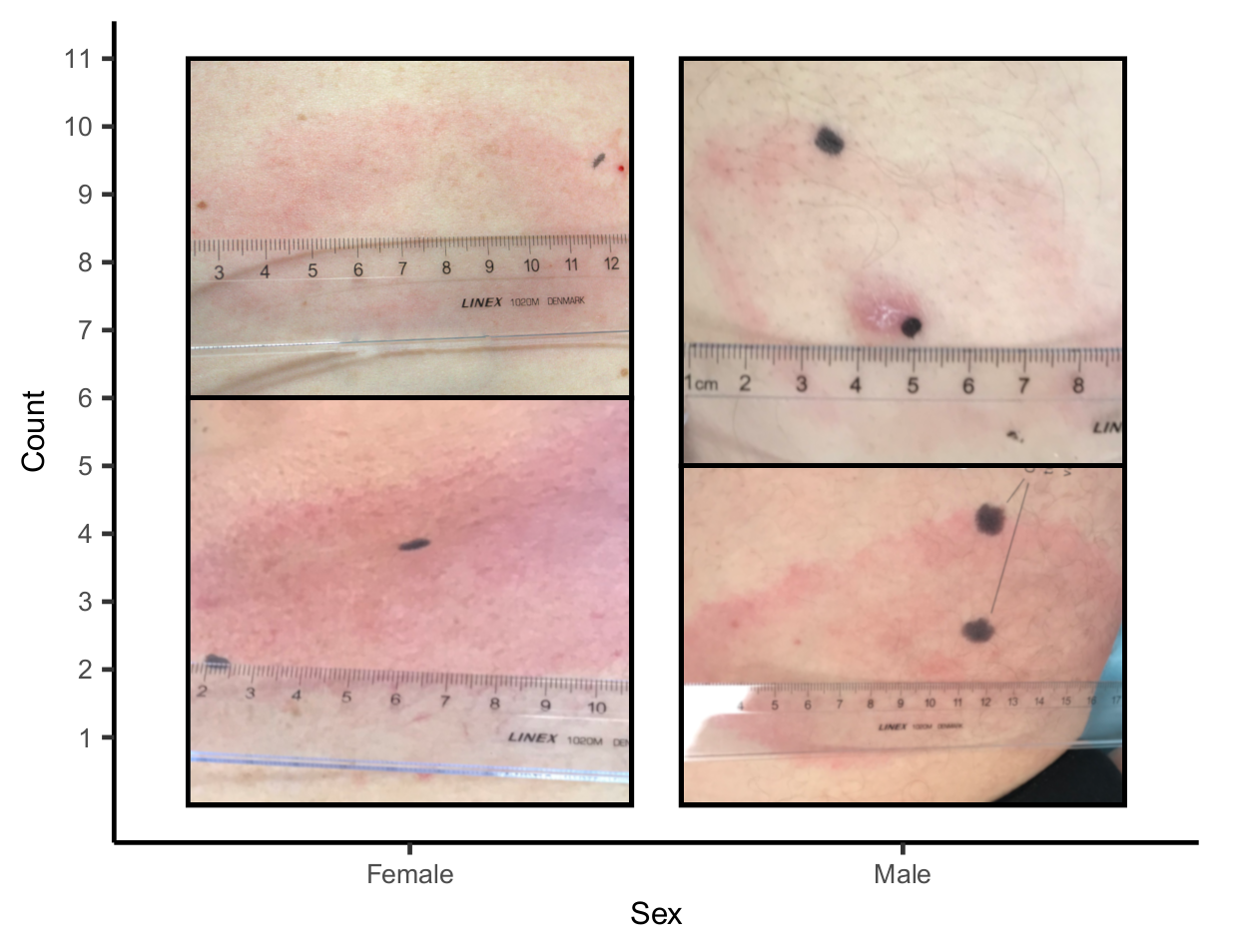
**Figure S2. Distribution of Erythema Migrans Morphology by Sex (n=22)
In this study erythema migrans morphology is evenly distributed between male and female sex. Annular erythema migrans are in the upper field, and homogeneous in the lower. Interestingly, both patients infected with B. garinii exhibited homogeneous erythema migrans. Previous studies have suggested that women and B. garinii infections are more likely to be associated with homogeneous erythema migrans. Black spots in the photographs are marked locations of biopsies.

##
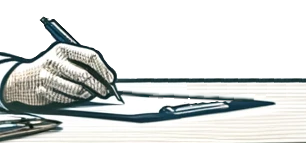

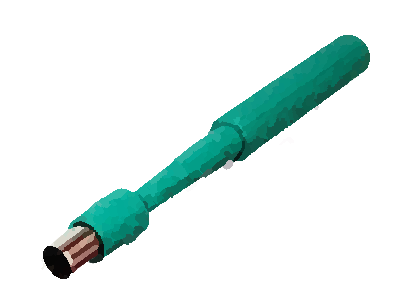

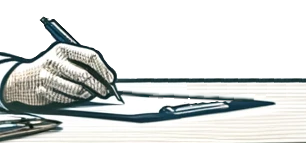

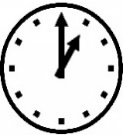

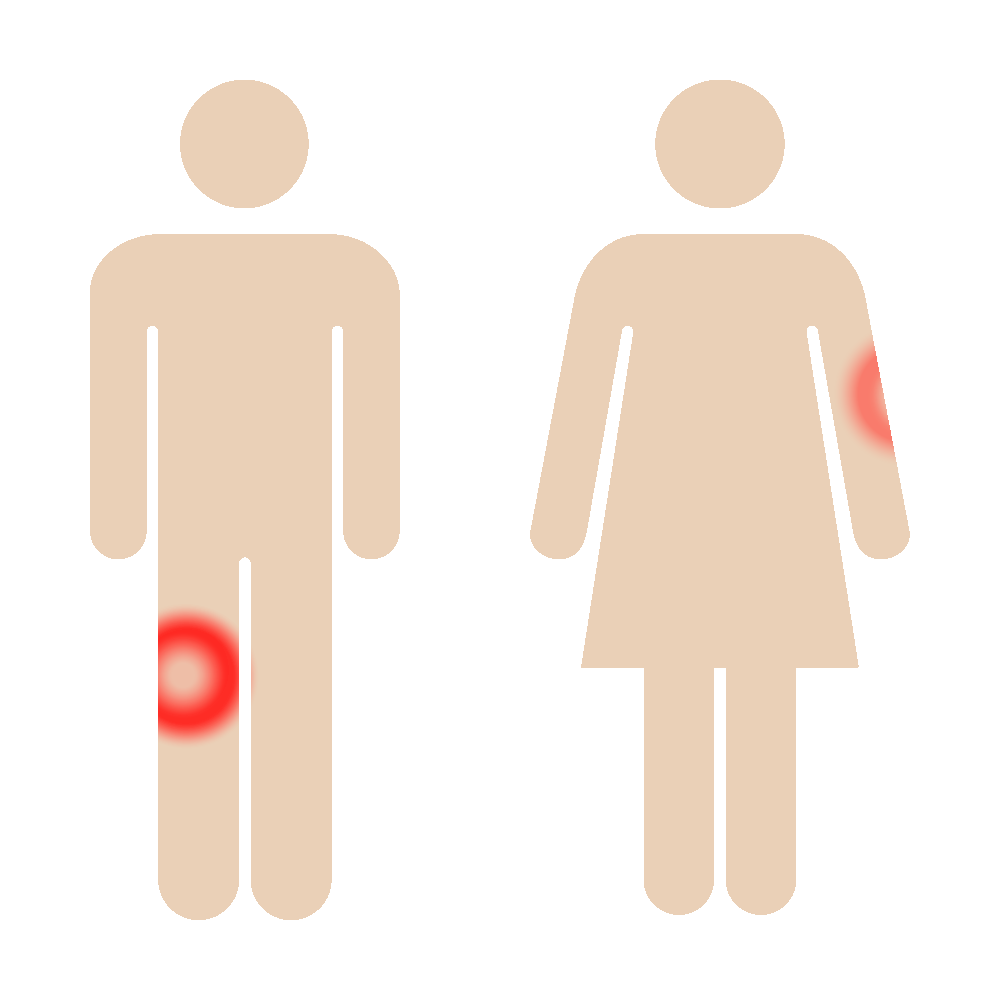
Figure S3. The schematic outline of the EMBio study


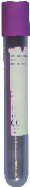

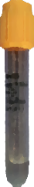

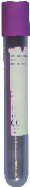

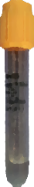


1) Patients clinically diagnosed with erythema migrans are sampled through up to three biopsies from multiple sites, along with blood samples. The physician documents symptoms and takes a photograph of the rash, while the patient completes a questionnaire.

3) Biopsies and blood samples are analyzed by molecular and serological detection methods.

2) After one month, the patient completes a follow-up questionnaire, and additional blood samples are collected.

##
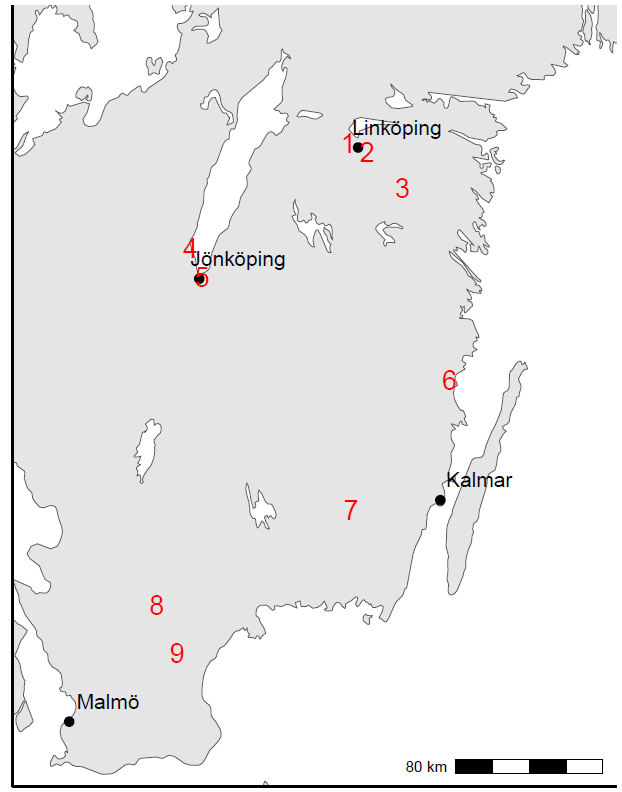
Figure S4. Map of Participating Outpatient Clinics (2018 – 2022)

EMBio study outpatient clinics (descending, geographic):

1. Primary Healthcare Center Kärna, Linköping, Region Östergötland
2. Primary Healthcare Center Ekholmen, Linköping, Region Östergötland
3. Primary Healthcare Center Åtvidaberg, Åtvidaberg, Region Östergötland
4. Primary Healthcare Center Bra Liv Habo, Habo, Region Jönköping County
5. Department of Infectious Diseases, County Hospital Ryhov, Jönköping, Region Jönköping County
6. Primary Healthcare Center Blå Kusten, Oskarshamn, Region Kalmar County
7. Primary Healthcare Center Emmaboda, Emmaboda, Region Kalmar County
8. Primary Healthcare Center Solbrinken, Hässleholm, Region Skåne
9. Primary Healthcare Center Tollarp, Tollarp, Region Skåne

The map was generated using R ([www.r-project.org](http://www.r-project.org), v4.5), Tidyverse ([www.tidyverse.org](http://www.tidyverse.org), v2.0.0) package with public domain map data from Natural earth ([www.naturalearthdata.com](http://www.naturalearthdata.com)).

| Table S5. Summary of the Questionnaires Questionnaire 1 — Completed by physician upon inclusion in the study | |
| --- | --- |
| Q1.1 | Anamnesis |
| a | Has the patient measured fever? |
| b | When was the EM discovered? |
| c | Has the EM grown? |
| d | Known tick bite? |
| e | Other symptoms i.e. myalgia, arthralgia, headache? |
| f | Other skin manifestations? |
| g | Novel neurological manifestations? |
| h | Other |
| Q1.2 | Physical examination |
| a | Description of EM |
| b | Lymphadenopathy? |
| c | Other |
| Questionnaire 2 — Completed by the patient upon inclusion in the study | |
| Q2.1 | Which date did you discover the rash? |
| Q2.2 | Do you know if you were tick-bitten at the site of the rash? |
| Q2.3 | If you answered yes on Q2.2, please answer the following questions: |
| a | When were you tick-bitten? |
| b | On which geographic location were you tick-bitten? |
| c | Where had you been? City / Town / Archipelago / Other |
| d | When was the tick removed? |
| e | For how long you think the tick fed? |
| f | Where was the tick? |
| g | Did you remove the entire tick? |
| h | Have you been previously tick bitten this season? If yes, how many times? |
| Q2.4 | Have you been previously treated for any tick-borne infection? |
| a | erythema migrans, date |
| b | other borrelia infection, date |
| c | anaplasmosis, date |
| d | tick-borne encephalitis, date |
| e | Did you receive any medical treatment for the infections above? If yes, which? |
| Q2.5 | Do you have any of the following medical conditions? |
| a | Allergies |
| b | Asthma |
| c | Diabetes |
| d | Tumor |
| e | Other skin condition |
| f | Other |
| Q2.6 | Have you undergone organ transplantation? |
| Q2.7 | Do you have any medications? If yes, which? |
| Q2.8 | Do you have any of the following symptoms? |
| a | Headache |
| b | Fatigue |
| c | Fever |
| d | Neck stiffness |
| e | Loss of appetite |
| f | Nausea |
| g | Weight loss |
| h | Vertigo |
| i | Attention difficulties |
| j | Radiating pains |
| k | Myalgia |
| l | Paraesthesia |
| m | Sonophobia |
| n | Photophobia |
| Questionnaire 3 — Completed by the patient upon one month follow-up | |
| Q3.1 | Have you had any additional tick-bites since during the previous month? |
| Q3.2 | Has the rash changed? If yes, how? |
| Q3.3 | Do you feel different now compared to before you had the rash? If yes, how? |
| Q3.4 | Have you experienced any of the following symptoms during the last month? |
| a | Headache |
| b | Fatigue |
| c | Fever |
| d | Neck stiffness |
| e | Loss of appetite |
| f | Nausea |
| g | Weight loss |
| h | Vertigo |
| i | Attention difficulties |
| j | Radiating pains |
| k | Myalgia |
| l | Paraesthesia |
| m | Sonophobia |
| n | Photophobia |
| o | Comments? New, improved, worsened symptoms? |
| Q3.5 | If you experienced symptoms in Q3.3 or Q3.4, did you seek healthcare for these? If yes, where and when? |

## S6. Protocol for assessing photographs in the EMBio study

Suspected tick bite?

yes no

Homogenous skin rash?

yes no

Annular skin rash?

yes no

Size (cm):

Assessment:

Tick bite reaction Erythema Migrans Other diagnosis

Comment:
